# Differentiation and displacement: Unpicking the relationship between accounts of illness and social structure

**DOI:** 10.1057/sth.2014.6

**Published:** 2014-06-11

**Authors:** Barry J Gibson, Ninu R Paul

**Affiliations:** a Unit of Dental Public Health, School of Clinical Dentistry, University of Sheffield, S10 2TA, UK

**Keywords:** illness accounts, systems theory, Luhmann, semantic analysis

## Abstract

This article seeks to unpack the relationship between social structure and accounts of illness. Taking dentine hypersensitivity as an example, this article explores the perspective that accounts of illness are sense-making processes that draw on a readily available pool of meaning. This pool of meaning is composed of a series of distinctions that make available a range of different lines of communication and action about such conditions. Such lines of communication are condensed and preserved over time and are often formed around a concept and its counter concept. The study of such processes is referred to as semantic analysis and involves drawing on the tools and techniques of conceptual history. This article goes on to explore how the semantics of dentine hypersensitivity developed. It illustrates how processes of social differentiation led to the concept being separated from the more dominant concept of dentine sensitivity and how it was medicalised, scientised and economised. In short, this study seeks to present the story of how society has developed a specific language for communicating about sensitivity and hypersensitivity in teeth. In doing so, it proposes that accounts of dentine hypersensitivity draw on lines of communication that society has preserved over time.

## Introduction

Exploring accounts of illness as a social and political phenomenon has been a prominent pre-occupation of the sociology of health and illness. Sociologists have helped to establish the significance of these accounts in a changing society (Bury, 1982, 1991, 2001; Hyden, 1997; Crossley, 1998). In addition, the literature has focused on how understanding accounts of illness can help patients and physicians (Anderton *et al*, 1989; Pinder, 1995; MacRae, 1999) and how they can enable society to observe the consequences of different illnesses and their treatment (Locker and Kaufert, 1988; Anderton *et al*, 1989; Pinder, 1995; Crossley, 1998; Bury, 2001). Pierret (2003) stated that one of the key remaining challenges for those working on the problem of illness experience was to:

define a paradigm and methodology for handling the problems related to the social structure. This entails working out theories about the interrelations, reciprocal effects and feedback between subjectivity, cultural factors and social structure. (p. 16)

Although the literature at that time did have some examples of attempts to address this aspect of illness experience, it was clearly felt that this subject required more sustained exploration. Attempts to explain the relationship between ‘subjectivity, cultural factors and social structure' have involved the use of numerous concepts. Some have used the concept of ‘contingent narratives' (Bury, 2001), others have used concepts such as morality, ‘the social', culture and ideology (Herzlich, 1973; Bury, 1982, 2001; Cornwell, 1984; Riessman, 1990; Williams, 1993; Kelly and Dickinson, 1997; Pound *et al*, 1998; Ballard *et al*, 2001; Pierret, 2003; Gibson and Boiko, 2012). In what follows we will briefly explore how each of these concepts have been used to help disentangle the relationship between social structure and illness experience before going on to outline the specific aim of this paper.

Bury (2001) is most notable for developing the concept of ‘contingent narrative' to articulate the focus individuals have on their beliefs about the causes of a disorder. Common themes of such discussions are that the medical system, culture and everyday life are significant sources of information for such narratives (Ballard *et al*, 2001; Bury, 2001; Pierret, 2003). Yet despite the identification of these as sources *how* this actually might work is not explained. A similar thing happens when morality is discussed as a significant source upon which accounts of illness rest (Herzlich, 1973; Bury, 1982, 2001; Cornwell, 1984; Riessman, 1990). Although it is widely regarded as a ‘source' for accounts of illness, there is little explicit discussion of *how* this happens. This is important because there is a tendency within the literature for many of the concepts to overlap and for some to be seen as a subset of others. For example, Bury (2001) in a commentary on the subject of illness narratives explores how Williams (1993) uses the concept of morality in ‘chronic illness' and how this is developed in order to locate patients' stories ‘within a cultural framework'(Bury, 2001, p. 275). Is morality a part of culture? In some respects we might see it this way but then is culture itself also a horizon of meaning? Does it subsequently encapsulate other aspects of meaning?

Bury (2001) provides some solutions to the problem although this is not the direct goal of his review. He states, while drawing on the work of Gellner (1992), that narrative can be seen as an important resource, a way of constituting an infinite ‘reservoir of meaning' with the goal of making sense of illness and its impacts (Bury, 2001, p. 264). He shows, drawing on the work of Kelly and Dickinson (1997), that culture can contribute to illness narratives by providing a series of frameworks (genres) that can then act as a ‘cultural resource' upon which people can draw when making sense of illness. In doing so, culture is both a restraint and an enabling resource (Gellner, 1992; Kelly and Dickinson, 1997; Bury, 2001).

The problem of the relationship between society and the experience of illness has also been explored within the Marxist tradition. Anderton *et al* (1989) uncovered how the experiences of White middle-class families contrasted with those from Chinese families living under conditions of deprivation. The concept of ‘normalisation' was mobilised as an ideological resource in order to obtain better outcomes for children from White middle-class backgrounds. In this sense, the relationship between the experience of illness and the accounts given to understand, explain and act on this experience is both social and political (Herzlich and Pierret, 1985; Anderton *et al*, 1989).

More recently writers such as Gibson and Boiko (2012) have explored how the work of Niklas Luhmann might be used to contribute to the wider understanding of the relationship between society and accounts of illness they argued that experiences of illness are narrated through ‘the voices and semantics of different systems' (ibid., p. 166). Their study sought to explore what these semantics might be in relation to the everyday impact of dentine hypersensitivity. Of the key distinctions that they uncovered the most important seemed to be that it was a ‘non-problem problem'. This peculiar distinction referred to the fact that while dentine hypersensitivity was problematic, it had extensive and often difficult everyday impacts, it was not a problem in general. It was not a serious problem for the dentist and it was something that was not a major problem in everyday life. It was simultaneously a problem and not a problem. Gibson and Boiko's (2012) interest was in the sociological significance of this distinction. They stated, in contrast to Bury (2001), that:

Rather than a reservoir of meanings being made available we have a series of systemic distinctions that condition the meaning of dentine sensitivity. In short, society's semantics serve to condense and stabilise meaning so that we know what to expect under certain conditions. (Gibson and Boiko, 2012, p. 183)

They went on to speculate that perhaps the distinctions they had uncovered bore some relationship to particular ‘social systems' such as science or medicine but they were unable to tell if this was the case from their interview data. Their data only served to direct attention to the content of the pool of meaning, they could not tell us *how these distinctions may have developed*. This brings us to the theoretical background for this article.

## Theoretical Background: Sense-Making, Narratives of Illness, Semantic Displacement and Social Structure

As we have seen this article takes as its starting point that narratives of illness are attempts at ‘making sense' of illness. In some respects this is not much different to that which can be readily determined from a cursory glance at this field of research (Bury, 1982; Williams, 1993, 2000; Bury, 2001; Pierret, 2003). Yet the approach we would like to propose goes further. We would like to argue that by adopting the tools of Luhmann's (1995) social systems theory this ‘sense-making' process should be seen to have a relationship to more general processes of ‘sense-making' that happen in a virtual plane (Luhmann, 1995; Farias, 2013). What this means is that the sense of what is being said in a communication about illness is not contained directly in narratives of illness but is displaced somewhere else. This other place is referred to as the virtual plane. Luhmann's focus has been on the abstract processes that shape this virtual plane.

The virtual plane of sense-making is shaped by communications that happen over time. However, what is communication? For Luhmann (1995), communication is the unity of three things: information, utterance and understanding. It is not the transmission of a message or content from a sender to a receiver and as a consequence does not travel through space and time. It is an event that occurs and as soon as it happens it disappears (Clam, 2000). The key event, in communication is how understanding is selected. For example, someone might grin or wince when someone else talks of their illness experience. What they do in response has meaning for that experience as it is communicated. Communication ‘happens retroactively, in the moment of understanding' (Farias, 2013, p. 7). It cannot be reduced to either information, utterance or understanding but combines these different elements.

Adopting this approach is similar to what Bury (2001) and others have been proposing. Here, however, we propose a set of concepts and a framework with which to understand sense-making, not only in communications about illness, but also how the virtual plane of sense-making developed. Sense-making, both in relation to an illness communication and also in relation to the background ‘virtual plane' is therefore something that is actualised out of a surplus of references to other possibilities of experience and action (Luhmann, 1995, p. 60).

Sense thus opens up a virtual world for communication, a world that does not encompass a collection of pre-existing things, but is rather an unlimited and unpredictable reservoir of lines of communication. (Farias, 2013, p. 8)

Narratives of illness are communications that actualise this medium in relation to illness and disease in so doing; however, they also simultaneously direct our attention to this virtual plane. In some senses they ‘displace' the problems that are being referenced within the narratives to this virtual plane. Yet the ‘sense-making' processes that build this virtual plane in the first place often remain hidden from view. However, how does such a virtual plane get constituted?

In systems theory the study of how society develops such pools of meaning is the study of social semantics (Luhmann, 1986, 1989, 1993, 2000). Åkerstrøm-Andersen (2003) states that semantics are:

characterised as the accumulated amount of generalised forms of differences (for example, concepts ideas, images and symbols) available for the selection of meaning within the systems of communication. In other words semantics are condensed and repeatable forms of meaning, which are at our disposal for communication. (Andersen, 2003, p. 87)

Semantics are established in society over long periods of time. In order for these to develop communications must link to each other. This linkage entails a fourth selection that must take place in communication. This refers to either the acceptance or rejection of the sense of communication (Luhmann, 1995). What matters is how previous communications are linked to what follows (Schneider, 2000). Mapping how this happens is termed semantic analysis. Before we move on to what this entails a few further points are necessary.

Communication linkages tend to differentiate along different types of reference problem. If, for example, communications link around the problem of scarcity then these communications become economised. When they link around making collectively binding decisions they become politicised. When they link around, if something is an illness or a health condition, they are medicalised. These problems of reference cannot be deduced theoretically and they are not ahistorical. They are contingent historical products that develop from concrete situations in everyday life (Luhmann, 1990; Farias, 2013). These virtual linkages and problems are never resolved, they come and go as reference problems develop and disappear.

Virtual reference problems are biased. They have a positive and negative code. Luhmann's historical studies sought to uncover genealogies of how highly specific problems of reference developed as ways of processing specific sense-making problems. In this perspective, the reference problems that are evident in narratives of illness (the social, morality, medicine and culture) are not simply amorphous pools of meaning. Rather, they should be seen as ‘forms of difference' composed of tensions and problems that can facilitate sense-making processes. We would suggest that narratives of illness point towards these pools of meaning and their underlying reference problems.

Finally, another important distinction that is relevant to the study of social semantics is the idea of concept and counter concept. As we have seen, semantics are generalised forms of differences that society has decided to preserve. How semantics achieve this is through the use of concepts. Concepts condense multiple meanings and expectations. The effect of this is to produce a horizon of meaning associated with the concept. These multiple meanings are always locked into the concept through its counter concept. Therefore, for example, a conceptual pair might be dentist\patient. The dentist might evoke a series of expectations such as, ‘small business owner', ‘health care professional', ‘sadist', ‘someone who fills teeth' to ‘someone who whitens teeth'. The concept is linked to a horizon of meanings that are never completely obvious but which have developed over time in association with the concept. The idea of a patient might also evoke various different expectations. Changes to the expectations of the patient such as ‘demanding', ‘lacking trust', ‘unwilling' or ‘nervous' will have an effect on the concept of the dentist. There is often a battle over such concepts and change in one concept can change the other.

As we have seen, Gibson and Boiko (2012) uncovered evidence that dentine hypersensitivity was commonly experienced and understood as a ‘non-problem problem'. Understanding how this became central to the accounts of illness they were studying involves establishing whether such sense-making processes are in some way related to a virtual plane of sense-making along with its underlying reference problems. This means going beyond Gibson and Boiko's (2012) analysis to explore how such a sense-making process might have developed in relation to dentine hypersensitivity, including diagnosing if there were any underlying reference problems. In what follows, we report on a study exploring the semantics of dentine hypersensitivity.

## Methodology

When it comes to the study of social semantics Luhmann was heavily influenced by *Begriffsgeschihte* or conceptual history (Luhmann, 1995, 1998, 1999; den Boer, 1998; Hampsher-Monk, 1998; Hampsher-Monk *et al*, 1998; Koselleck, 1998, 2002, 2004; Åkerstrøm-Andersen, 2003, 2011). This approach is inspired by Saussure's observation that there is a distinction between the synchronic and diachronic aspects of language. Hence, while a language might change over time, at any point in time it has a definite structure. The study of conceptual history tends to alternate between synchronic and diachronic analysis of a particular semantic field (Hampsher-Monk *et al*, 1998). Another important principle of conceptual history is not to identify the concept under study with any single word or words but that there will often be a range of terms designating the same underlying concept.

In the present study, this involved the use of three strategies deployed in two overlapping stages of analysis. First, we studied the semantic field *in the current literature* that meant analysing the range of terms (synonyms and antonyms) that form part of the broad vocabulary associated with the current understanding of dentine hypersensitivity. Second, we drew on the techniques of onomasiology and semasiology to establish the range of terms and references within the pool of meaning. Onomasiology is the ‘study of different terms available for designating the same or similar thing or concept' (Hampsher-Monk *et al*, 1998, p. 2). Semasiology ‘seeks to discover all of the different meanings of a given term' (ibid). Third, since an important aspect of communication and sense-making is the fourth selection of meaning, we explored how communications linked to each other and what this meant for the meaning of the concepts being studied. Our focus was on diagnosing how the chain of sense-making within the literature became constituted over time. In what follows, we describe how these techniques were deployed in the two overlapping stages of synchronic and diachronic analysis.

### Synchronic analysis

In the beginning, we used onomasiology and semasiology to explore the current professional literature on dentine hypersensitivity. Our initial analysis focused on establishing the synchronic meaning of dentine hypersensitivity through a careful analysis of definitions and discussions of the terms used to refer to the concept. We began by searching standard online databases including Web of Knowledge, PubMed and OvidSP using the terms ‘dentin*hypersensitivity', this search revealed a wide range of citations. The search results revealed a total of 749 citations in the Web of Knowledge; 905 in PubMed and 151 in Ovid Medline. Obviously not all of these articles were relevant for this study.

It is important to bear in mind that we were interested in the sense-making process related to the changing meaning of the concept within the professional literature. Although a great number of studies use the language and concepts associated with ‘dentine hypersensitivity' very few discuss the meaning of the concept. The term is often used to report the findings of *in vivo* or *in vitro* studies but nothing is really said about the condition beyond that. In addition, such studies often did not add to the changing pool of meaning other than by reporting whether a particular de-sensitising agent was useful or not. As a result, we narrowed the initial search by looking for those articles with ‘dentine hypersensitivity' and ‘review' in the title. This reduced our search to a detailed study of 75 articles. By scanning through these articles we found that many were not central to the definition and specification of the underlying problem or concept, indeed they often took for granted its meaning. They were also based on a smaller subset of key articles. These articles were therefore much more central to the current meaning of the condition. They were also more widely cited and as a result we could be sure they were central reference points for the current meaning of the concept. This reduction left us with 17 articles that became central to the *synchronic analysis* (see Table 1). As we can see the work of Dowell and Addy (1983), Pashley (1986, 1990) and Absi *et al* (1987) are central nodes in the literature, they are most widely cited and are central to the contemporary meaning of the term. Of particular note is another paper that provided a detailed history of the diagnosis and treatments for the condition (Rosenthal, 1990). This article was particularly useful as it reviewed other work going back to the nineteenth century and so provided us with a way into the historical literature.

When it comes to the current meaning of dentine hypersensitivity, the work of Dowell and Addy (1983) in particular highlighted the central problem of using the concept. They began by stating that:

The pain arising from exposed dentine, typically in response to chemical, thermal, tactile and osmotic stimuli, is varied in both frequency and severity. For most patients the pain, which typically follows instantaneously upon application of the offending stimulus, is short-lived and usually resolves immediately after withdrawal of the stimulus. Diagnostic difficulties are created since the symptoms do not differ from those which may be reported with dental caries and its associated pulpal changes. However, in the absence of other dental pathology, when such symptoms arise from the dentine exposed to the oral environment, the term ‘dentine hypersensitivity' is used to describe the condition. (Dowell and Addy, 1983)

They went on to describe the difficulty of distinguishing dentine hypersensitivity from dentine sensitivity:

To date no attempt has been made to clearly define the term ‘dentine hypersensitivity'. In fact, since for the majority of sufferers, pain from exposed dentine only occurs on the application of the stimulus, the term ‘dentine sensitivity' could equally well be applied … This situation has arisen for several reasons. There is a dearth of information concerning the pulp changes, if any, associated with dentine hypersensitivity. Most studies correlating clinical signs and symptoms with pulpal pathology have been concerned with dental caries and its sequelae … Dentine hypersensitivity is thus, perhaps, more a symptom complex than a true disease and the severity of the pain or the patient's interpretation of this, appears to determine whether treatment is sought. (Dowell and Addy, 1983, pp. 342–343).

It was clear at that time that dentine hypersensitivity suffered from problems of definition and that the concept seemed to be either confused or associated with a related problem; that of dentine sensitivity. At this point we were wondering if we had an example of a concept and its counter concept. *Diagnosing how these terms became retro actively connected to each other, how they came to be central to the pool of meaning, including how they provided a surplus of references for the concept of dentine hypersensitivity became the focus of the second stage of analysis.*

### Diachronic analysis

Now we had established that there was a link between two closely related concepts, dentine sensitivity and dentine hypersensitivity, we sought to trace the emergence of these concepts over time. We did this by mapping all the associated terms that had been deduced from the onomasiological and semasiological analysis of the literature (Hampsher-Monk *et al*, 1998). In doing so, we traced the earliest reference we could find to words and terms we knew had been associated with the concepts of dentine hypersensitivity and dentine sensitivity (for example, ‘sensibility of dentine', ‘sensitiveness of dentine', ‘dental neuralgia'). This was achieved by paying close attention to the texts and looking back through the citations that formed part of the synchronic analysis and reading those papers too. Thus, for example, we uncovered a citation to the work of Gysi (1900) who referred to earlier sources and concepts. *Our approach was to identify key points in time when the underlying concepts and distinctions in dentine hypersensitivity changed.* In this respect we follow Åkerstrøm-Andersen's (2011) approach in tracing several different movements in the meaning of dentine hypersensitivity. This included looking to establish how these two concepts became entangled in the sense-making process and looking at changes in the underlying reference problems (Luhmann, 1995; Åkerstrøm-Andersen, 2011). In what follows, we present the findings of this diachronic analysis. We do this because a presentation of the synchronic analysis is less sociologically interesting and there is a need to conserve space.

## Differentiation and Displacement: The Emergence of Dentine Hypersensitivity

Nettleton (1992), following Foucault (1963, 1998) argues that the mouth became separated from the body *through its creation as an object of knowledge and governance* (Nettleton, 1992). This process involves opening up the mouth to the clinical gaze, understanding its colours, textures and cell arrangements. It also involves understanding how to manage these through the techniques of surveillance and governance (Foucault, 1963; Nettleton, 1992). In this study, what we uncovered is that embedded within this general ‘sense-making' process are important processes of *differentiation* (Luhmann, 1982; Luhmann, 1990). This process involves distinguishing different qualities of sensation and pain in thinking feeling subjects.

Concerns about the underlying problem of sensitive teeth date back thousands of years. Although, the earliest reference we could find to anything talking specifically about the sensitivity of teeth was in 1827 when Perry discussed the ‘sensibility of the tooth' as a consequence of the loss of enamel in response to acid attacks. This loss of enamel was said to occur in the early stages of disease (Perry, 1827). The discipline continued to distinguish and differentiate the underlying mechanisms of this ‘sensibility' (Blandy, 1851). Hence, for example, it was proposed that small holes called ‘tubules' ran from the circumference of the tooth to the centre and these were filled with fluid secreted from the tooth pulp. It was suggested that the ‘sensibility of the tooth' was a consequence of water moving in and out of these ‘tubules' and this was conceptualised as a ‘hydrostatic pressure'. This movement could also happen as a result of various stimuli such as cold water, rubbing or scraping and contact with certain substances (Discussion at American Dental Convention, 1856; Harris, 1885). However, the differentiation process really begins to take shape when in 1900 a distinction began to develop between ‘sensibility' and ‘sensitiveness'. The sensitiveness of dentine:

… is only of a secondary nature, and is not physiologic. The physiological sensibility of dentine is sufficiently provided for by the pulpa and the periosteum, so that the supply of nerves in dentine would be superfluous. If the dentinal canalicules contained nerves, the progress of caries would be painful, which is not the case as long as the pulp is not attacked. (Gysi, 1900, pp. 865–866).

These different concepts, the ‘sensitiveness of dentine' and the ‘physiological sensibility of dentine' concerned *differentiating* biological mechanisms and therefore producing *different* sensations of pain (Harris, 1885). The differentiation process was therefore producing different reference points for communication about sensations in teeth as a result of different underlying pathologies. It was also differentiating the sensations that occurred during *dental treatment*. It is obvious that sensitivity was getting in the way of the work of dentists. For example, the principal source of ‘sensibility' of dentine, was found to be the stimulation of the pulp as a consequence of the movement of fluid through tubes that run through dentine. These tubes, called ‘canalicules', were filled with an ‘aquous content' so that:

A pressure or drawing exercised upon the aquous content of a dentinal canalicule that opens into a carious cavity is directly transmitted to the other end of the dentinal canalicule where it is loosely closed by the odontoblasts and then the odontoblasts which are abundantly interwoven with nerves feel the pressure or drawing as a sensation of pain. (Gysi, 1900, p. 866).

The ‘pressure' being referred is the sensations that occur in response to the ‘drawing' or excavation of dental caries (disease) *in dental treatment* (Gysi, 1900). The quality of these sensations became perhaps *the* central reference problem for dentistry marking the point at which the ‘sensitiveness of dentine' was *differentiated* (Luhmann, 1995) from the ‘sensibility' of teeth. This differentiation process is important. An early sign of dental disease was understood to be enamel loss and this became understood as the main cause of the *sensibility* of dentine (Perry, 1827). By the turn of the twentieth century, a key cause of ‘sensitivity of dentine', was dental treatment (Gysi, 1900). As we can see from Table 2, before this point ‘sensitivity' *included* incipient disease, dental treatment and chemical action (Discussion at American Dental Convention, 1856; Harris, 1885). The *differentiation* of ‘sensibility' from ‘sensitivity' resulted in different reference problems. One reference was to the clinical management of disease (sensitivity) and the other to a pain experience in the absence of disease (sensibility). Even though these were *differentiations* in the pool of meaning each of the various concepts remained tied to each other *because they needed to be distinguished from each other* and acted on in different ways.

The practical work of the clinic generated different forms of sensitivity referring to different underlying reference points for problems of diagnosis and management. These findings are very similar to the work of Mol (2002) in ‘The body multiple' that draws on a material semiotics and actor network theory. In this work the disease, atherosclerosis, is ‘enacted' through different practices in different parts of the hospital. In one place, atherosclerosis is the thickening of the intima visible under a microscope. In another it becomes an inability to walk a certain distance without pain. Mol (2002) indicates several key things. First, diagnosis is the practical management of the body, something is done with the body. It involves poking, touching dicing and manipulating the body. Second, diagnosis involves material relationships and this enables particular aspects of the body to become intelligible. Third, multiple practices enact disease in multiple forms which in turn are called multiple realities. In this study, it was primarily processes of *differentiation* (Luhmann, 1995) that led to different concepts of sensitivity, sensitiveness and sensibility, which were nonetheless in some way all related to each other. This process of *differentiation* contributed to the production of different lines of observation and communication about sensitivity in teeth. They also contributed to the pool of meaning that was developing around sensitivity.

These differentiation processes were critical because they prepared the way for the eventual emergence of the concept of dentine hypersensitivity. At this time ‘sensitive dentine' was distinguished from ‘hypersensitive dentine':

… term sensitive dentin applied to this condition is a misnomer; all vital dentin is sensitive, and its degree of sensitivity differs markedly in individuals; it is only when hypersensitivity is observed that the condition becomes pathological. Hypersensitivity of dentin may be defined as such a degree of sensitiveness as interferes with the proper excavation and shaping of a carious cavity; or which, in the absence of dental ministrations, causes painful symptoms, as a rule reflected about neighbouring parts. (Burchard, 1915, p. 393).

In this quotation ‘sensitive dentine' is considered ‘normal', hypersensitivity of dentine ‘pathological'. Yet ‘hypersensitivity of dentine' is pathological *because it interferes with dental treatment*. In this instance, one concept, sensitive dentine becomes a ‘normal' problem to be managed. This concept is propped up by another concept, hypersensitivity, which became an ‘abnormal' overreaction. Dentine sensitivity became a normal problem to be managed as part of the practical management of teeth in the clinic, ‘dentine hypersensitivity' an unmanageable condition, a pathological overreaction. We would suggest that dentine hypersensitivity became a counter concept to dentine sensitivity.

As we have already noted, these ideas relate to challenge coming from the emerging sociology of disease. In this approach, the challenge has been for sociology to develop a nuanced understanding of the physiological diversity of different conditions (Mol, 2002; Timmermans and Haas, 2008). Following this approach Gardner *et al*, (2011) have proposed that specific ‘diseases involve specific and often multiple disease diagnostic practices, which, in turn, can generate particular experiences of the body' (p. 849). Here we find that the sensitivity of teeth developed very different meanings. These differences in meaning referring to very different problems. First, the classification of sensitivity into different categories happened because of the practical problem of making sense of and managing sensitivity during the work of the dental clinic. Second, the classification of different sensations as sensitive or hypersensitive enabled dentistry to focus on what would be its central concern; the management of sensitivity during dental treatment. This emphasis had an impact on the status of dentine hypersensitivity that remained a highly unstable and indeed controversial category:

… there are no nerves in dentine … consequently, no sensation in dentine. There is no hypersensitivity of dentine. (Hopewell Smith, 1924, p. 76).

Therefore, not only is the body a site for multiple lines of investigation but these jostle for position, push up against one another producing different emphases and generating diverging plans of action. They change the pool of meaning by enabling a wider repertoire for making sense of various sensations in teeth. Even though dentine hypersensitivity was an unstable category efforts continued to try and understand the condition. There was, for example, a series of communications in the literature seeking out the underlying mechanisms of dentine hypersensitivity (see Table 2). Several mechanisms were proposed for the condition: on the one hand, it was claimed that the nerves in dentine were acting as pain receptors; on the other, osmotic changes in the fluid filled dentinal tubules caused activation of pain receptors in the pulp, and finally the tubules themselves were said to be part of the sensory mechanism (Ambrose, 1943). No individual hypothesis was refuted and this left dentine hypersensitivity more or less with the appearance of a medically ‘unexplained symptom'^1^ (Undeland and Malterud, 2002; Nettleton, 2006; Jutel, 2010; Greco, 2012).

A critical stage in the process of differentiating dentine hypersensitivity from dentine sensitivity occurred in a series of studies by Brännström (1961, 1962a, 1962b, 1963a, 1963b, 1965, 1986) (see Table 2) involving experiments looking at the condition in relation to various stimuli (Johnson and Brännström, 1974). As a consequence, the ‘hydrodynamic theory' was confirmed as the best explanation for the continued presence of sensitivity in the absence of other causes (Brännström, 1963a). These developments eventually culminated in a consensus statement in Canada that dentine hypersensitivity was ‘a short, sharp pain arising from exposed dentin in response to stimuli typically thermal, evaporative, tactile, osmotic or chemical and which cannot be ascribed to any other form of dental defect or disease' (Canadian Advisory Board for Dentine Hypersensitivity, 2003). Yet despite this, dentine hypersensitivity remained conceptually bound to dentine sensitivity. It could only be diagnosed *after dental pathology had been ruled out.* This ‘differential diagnosis' has been shown to be common in other ‘medically unexplained' conditions such as neuralgia (Nettleton, 2006; Jutel, 2010; Greco, 2012). However, unlike these conditions, where there is a lack of legitimacy in diagnosis, dentine hypersensitivity as a category has become relatively well established. However, how was this achieved? So far in our analysis we have uncovered how dentine hypersensitivity became established *as a line of communication in dental clinics*. In the next section, we will explore how this line of communication also involved exploring practical solutions to the problem.

## The Market and Dentine Hypersensitivity

So far our analysis has been focused on diagnosing the effects of the *differentiation* of separate points of reference for dentine sensitivity and hypersensitivity. As we shall see the differentiation of these points of reference had consequences for the solutions that were developed for each problem. The solutions to the problem of sensitivity and hypersensitivity took different forms because they were conceived of as different problems. These differences acted as reference points for communication about the various problems as well as the eventual solutions. Dentine sensitivity remained a central problem for dentistry because it interfered with dental treatment. It would eventually be managed through the use of local and general anaesthetics. Dentine hypersensitivity became something to be managed at home through the use of various ‘dentifrices'.

As stated previously the treatment of sensitivity goes back centuries but for our purposes the earliest references we could find to dentine sensitivity was 1825 (The Examiner Classifieds, 1825). By the 1920s, commercial preparations such as Sensitex, had become available. Rosenthal (1990) recorded around 104 different substances that were used as solutions to the problem of dentine sensitivity. These included asbestos, anodyne cement, carbolic acid, and morphine, oleate of cocaine and quinine sulphate among other things (Rosenthal, 1990, p. 406). Rosenthal (1990) also explained how a report by Pawlowska and Jego Znaczenie (1956) detailed that strontium chloride could produce a good effect on dentine hypersensitivity (Pawlowska and Jego Znaczenie, 1956; Rosenthal, 1990). On the basis of this Sensodyne toothpaste was ‘formulated with strontium chloride hexahydrate' (Rosenthal, 1990, p. 417). Eventually ‘Sensodyne', a toothpaste for dentine hypersensitivity, was introduced in the United States in 1961. Sensodyne is not the only dentifrice that has been developed for dentine hypersensitivity, however. Emoform toothpaste existed in Switzerland in the late 1940s to the early 1950s containing formaldehyde, calcium carbonate, magnesium carbonate and a mineralising salt composed of sodium bicarbonate, sodium chloride, potassium sulphate and sodium sulphate (Rosenthal, 1990). This substance was eventually licensed to Thomas Leeming Co. and sold in the United States as Thermodent Toothpaste. Indeed this toothpaste was the leading brand until the early 1960s when Sensodyne eventually surpassed it in terms of sales. During this time, research into the effectiveness of these toothpastes was being sponsored (Fitzgerald, 1956).

We would like to suggest that these processes had several implications. First, the production of various remedies produced different solutions that reflected how the underlying problems were conceived. These differentiations produced different lines of communication and action that then enabled dentists and patients to investigate and manage sensitivity in different ways. The differentiation of dentine sensitivity and dentine hypersensitivity was not only producing the underlying diagnostic categories but it had an effect on the solutions that were envisaged. Although Mol (2002) observed the same condition having multiple realities, here the differentiation of the same sensations produced different conditions and solutions. Dentine hypersensitivity can only be established through a differential diagnosis and as such it remains dependant on the elimination of dentine sensitivity as a condition. This had consequences for the experience of dentine hypersensitivity. It became a problem that dentists tended to avoid. Thus, on one level it became a ‘non-problem problem' (Gibson and Boiko, 2012) because it was not a problem for the dentist.

Yet the production of commercial solutions to the problem of dentine hypersensitivity also *produced* it as a condition to be managed *at home* through the use of specialist dentifrices. In doing so, dentine hypersensitivity became a ‘non-problem problem' precisely because it could be easily managed. The suggestion is that while considerable legitimacy flows from being defined as a medical problem in keeping with the work of Nettleton (2006) and others (Jutel, 2010; Greco, 2012), legitimacy can also be derived from the market. This source of legitimacy, however, appeared to generate a different horizon for the meaning of the condition. It means that when we talk of dentine hypersensitivity we talk of a health condition rather than a disease, something that is trivial rather than problematic. In contrast to medically unexplained symptoms (Nettleton, 2006; Jutel, 2010; Greco, 2012), patients have a label that tells them how they can speak about their condition and more importantly what they can do about it.

## Discussion

The starting point for this article was that there is a ‘pool of meaning' (Bury, 2001) that acts as a resource from which accounts of dentine hypersensitivity draw. What it suggests is that with respect to dentine hypersensitivity this pool of meaning is not simply a surplus of references but that it is composed of a series of different ‘lines of communication' (Luhmann, 1995). The range of concepts and lines of communication developed over the last 186 years to enable society to not only make sense of sensitivity in teeth but to manage this sensitivity in different ways. When communication about dentine sensitivity happens it refers in some way to this pool of meaning in *an attempt to make* sense of the underlying sensation. This attempt to make sense seeks to tease out whether or not the sensations are a consequence of pathology or if they are relatively ‘trivial'. This ‘differentiation' process then resulted in the same underlying symptom being understood through different meanings and solutions. Dentine sensitivity became sensations that resulted from dental disease and required management during dental treatment. Dentine hypersensitivity is an above normal reaction to various stimuli that could be ‘remedied' through the use of different toothpastes. The narratives of dentine hypersensitivity in Gibson and Boiko's (2012) study refer to this pool of meaning and operate through these distinctions.

By adopting the approach of conceptual history we have sought to tease out how one ‘conversation in mankind' (Scholz, 1998) has sought to distinguish different qualities of sensitivity in teeth. Throughout this account we have suggested that the development of dentine hypersensitivity as a concept involved the constant displacement of various underlying reference problems. Thus, for example, dentine hypersensitivity when it first emerged was developed to articulate abnormal reactions to dental treatment. By focusing on normal sensitivity dentists left these overreactions to the patient to be managed. This displacement to the outside of the work of the clinic had consequences for hypersensitivity. It remained an unstable concept and lacked legitimacy. Yet despite this displacement it remained a persistent problem that required an explanation and a solution. This is why we can see other communication that ‘scientised' the underlying reality and others that ‘marketised' the solution in the form of sensitive toothpastes. Behind these developments, it might be suggested, are the effects of functional differentiation. Dentine hypersensitivity was simultaneously medicalised, scientised and economised (Luhmann, 1995). It suggests that what Gibson and Boiko (2012) uncovered in their narratives was not simply a distinction that had its roots in the medical system, but rather a distinction that got its meaning from the varied effects of this broad social process.

The story we have presented here is the story *behind* the way society has developed a specific language for communicating about sensitivity in teeth. It is the task of conceptual history to pay attention to which groups take charge of concepts and the biases this produces (Scholz, 1998). In this story, dentistry and dentists took charge of sensitive teeth, this became their domain. In doing so, they set boundaries around what was normal and what was abnormal. The discipline focused on controlling, studying and treating the causes of ‘normal', ‘pathological' sensitivity, leaving dentine hypersensitivity to become a peculiar abnormality, an irritation to the work of the clinic. Yet this story is not simply about how a particular clinical gaze operated. As we have seen interest in dentine hypersensitivity did not stop. Over the period of 186 years remedies were developed for both the problem of dentine sensitivity and dentine hypersensitivity. These developments had an effect on the meaning of dentine hypersensitivity. It became a problem with a relatively ‘simple' solution; use toothpaste. The problem, it seems, was defined, displaced, trivialised and transformed into a non-problem problem.

## Figures and Tables

**Table 1:** Synchronic analysis: Starting references

*Date*	*Paper details*	*Citations*
*1976*	Harris, R and Curtin, J.H. (1976) Dentine hypersensitivity. *Australian Dental Journal* 21(2): 165–169	**5**
*1983*	Dowell, P. and Addy, M. (1983) Dentine hypersensitivity – A review: Clinical and *in vitro* evaluation of treatment agents. *Journal of Clinical Periodontology* 10: 351–363	**172**
*1985*	Berman, L.H. (1985) Dentinal sensation and hypersensitivity: A review of mechanisms and treatment alternatives. *Journal of Periodontology* 56(4): 216–222	**18**
*1986*	Pashley, D.H. (1986) Dentine permeability, dentine sensitivity and treatment through tubule occlusion. *Journal of Endodontics* 12(10): 465–474	**154**
*1987*	Absi, E.G., Addy, M. and Adams, D. (1987) Dentine hypersensitivity – A study of the patency of dentinal tubules in sensitive and non-sensitive cervical dentine. *Journal of clinical Periodontology* 14: 280–284	**137**
*1987*	Absi, E.G., Addy, M. and Adams, D. (1987) Dentine hypersensitivity – The development and evaluation of a replica technique to study sensitive and non-sensitive cervical dentine. *Journal of Clinical Periodontology* 16: 190–195	**37**
*1990*	Rosenthal, W. (1990) Historic review of the management of tooth hypersensitivity. *The Dental Clinics of North America*. 34(3): 403–427	**14**
*1990*	Pashley, D.H. (1990) Mechanism of dentine sensitivity. *The Dental Clinics Of North America* 34(3): 449–469	**129**
*1990*	Addy, M. (1990) Etiology and clinical implications of dentine hypersensitivity. *The Dental Clinics Of North America* 34(3): 503–514	**65**
*1994*	Addy, M. and West, N. (1994) Etiology, mechanisms, and management of dentine hypersensitivity. *Current Opinion in Periodontology*: 71–77	**9**
*2000*	Addy, M. (2000) Dentine hypersensitivity: Definition, prevalence, distribution and etiology. In: Addy M. *et al* (eds.) Tooth wear and sensitivity-clinical advances in restorative dentistry. London, Martin Dunitz	**53**
*2002*	Addy, M. (2002) Dentine hypersensitivity: New perspectives on an old problem. *International Dental Journal* 52(5): 367–375	**51**
*2003*	Canadian Advisory Board for Dentine Hypersensitivity (2003): Consensus based recommendations for the diagnosis and management of dentine hypersensitivity. *Journal of Canadian Dental Association* 69(4): 221—226	6
*2005*	Addy, M. (2005) Tooth brushing, tooth wear and dentine hypersensitivity – Are they associated? *International Dental Journal* 55(4): 261–267	37
*2005*	Walters, P.A, (2005) Dentinal hypersensitivity: A review. *The Journal of Contemporary Dental Practice* 6(2): 107–117	35
*2006*	Bartold, P.M. (2006). Dentinal hypersensitivity: A review. *Australian Dental Journal* 51(3): 212–218	20
*2006*	Orchardson, R. and Gillam, D.G. (2006). Managing dentin hypersensitivity. *Journal of the American Dental Association* 137(7): 990–998	64

**Table 2:**
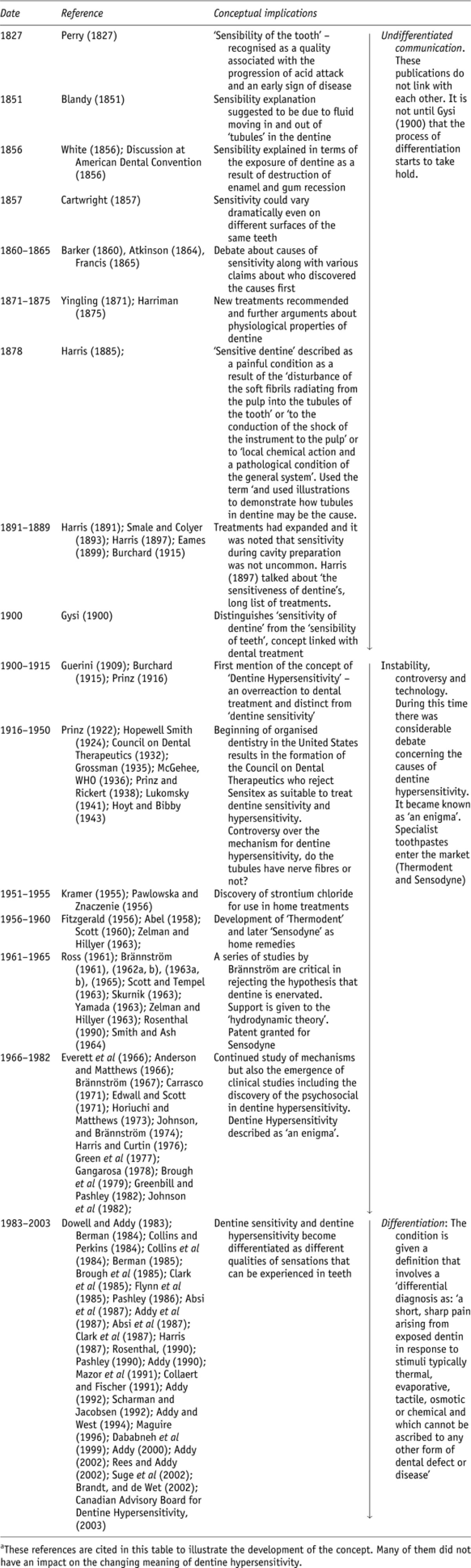
The differentiation of dentine sensitivity and dentine hypersensitivity^a^

## Footnotes

1 We would like to acknowledge one of the anonymous reviewers for suggesting that we consider this concept.
